# Hemostatic Properties of Aortic Root Preservation versus Root Replacement for Acute Type A Aortic Dissection: A Pooled Analysis

**DOI:** 10.3390/life14101255

**Published:** 2024-10-01

**Authors:** Dimitrios E. Magouliotis, Arian Arjomandi Rad, Alessandro Viviano, Aung Ye Oo, Andrew Xanthopoulos, Serge Sicouri, Basel Ramlawi, Thanos Athanasiou

**Affiliations:** 1 Department of Cardiac Surgery Research, Lankenau Institute for Medical Research, Main Line Health, Wynnewood, PA 19096, USA; sicouris@mlhs.org; 2 Division of Medical Sciences, University of Oxford, Oxford OX1 4BH, UK; arian.arjomandirad@medsci.ox.ac.uk; 3 Department of Surgery and Cancer, Imperial College London, London SW7 2AZ, UK; alessandro.viviano@nhs.net (A.V.); t.athanasiou@imperial.ac.uk (T.A.); 4 Department of Cardiothoracic Surgery, St. Bartholomew’s Hospital, London EC1A 7BS, UK; aungye.oo@nhs.uk; 5 Department of Cardiology, University of Thessaly, Biopolis, 41110 Larissa, Greece; andrewvxanth@gmail.com; 6 Department of Cardiac Surgery, Lankenau Heart Institute, Main Line Health, Wynnewood, PA 19096, USA; ramlawib@mlhs.org

**Keywords:** aortic dissection, ATAAD, aortic repair, root preservation, root replacement, valve sparing, Bentall

## Abstract

Objective: We reviewed the available literature on patients undergoing aortic repair for acute type A aortic dissection (ATAAD) with either aortic root preservation (RP) or root replacement (RR). Methods: Original research studies that evaluated short- and mid-term hemostatic properties of RP versus RR groups were identified, from 2000 to 2024. Intraoperative transfusions of red blood cells (RBCs), reoperation for bleeding, strategy of hemostatic sealing of the anastomosis in root repair following the reapproximation of the dissected layers of the aortic wall (with/without biological glue), and operative mortality were the primary endpoints. Postoperative morbidity and overall and reoperation-free survival at one and five years were the secondary endpoints. A sensitivity analysis was performed using the leave-one-out method. Results: Ten studies were included in the qualitative and quantitative synthesis, incorporating data from 6850 patients (RP: 4389 patients; RR: 2461 patients). Root preservation demonstrated a lower median transfusion of RBCs (WMD: −1.00; 95% CI: −1.41, −0.59; *p* < 0.01) and incidence of reoperation for bleeding compared to root replacement (OR: 0.67; 95% CI: 0.58, 0.77; *p* < 0.01). The majority of studies did not use biological glue in root repair to avoid the risk of an anastomotic pseudoaneurysm. No difference was found regarding postoperative morbidity, along with mid-term overall and reoperation-free survival. Conclusions: Root preservation without the use of biological glue during aortic repair is associated with enhanced hemostatic traits compared to the root replacement approach. A future well-designed Randomized Controlled Trial should further validate our outcomes.

## 1. Introduction

Acute type A aortic dissection (ATAAD) represents a cardiac surgery emergency associated with high rates of morbidity and mortality [1]. Patients with ATAAD tend to present with highly heterogeneous aortic root pathology that might involve the aortic root and the aortic arch to different extents, with or without the presence of aortic valve insufficiency. The main goal of the surgical treatment strategy is to restore the true lumen blood flow by resecting the primary intimal tear, along with replacing the ascending aorta and transverse arch. Nonetheless, decision-making is a much more complex task in cases of a simultaneous retrograde propagation of the dissection into the aortic root along with the presence of aortic valve insufficiency [2]. In this context, the aortic root management strategy might be either conservative, with aortic root preservation (RP) through neomedial reconstruction and commissural resuspension, or more aggressive with aortic root replacement (RR) [2]. One of the main dilemmas in choosing one of the two approaches is whether choosing the more aggressive approach of root replacement is associated with worse outcomes in terms of hemostasis and blood transfusion, thus affecting perioperative morbidity.

There is still an important debate whether either approach provides superior outcomes in terms of hemostasis, mainly due to the lack of a Randomized Controlled Trial (RCT). In fact, the short-term hemostatic traits and the mid-term durability of root-preserving aortic repair remain unclear. In this context, there is evidence suggesting that RP is associated with a higher incidence of reoperation [2]. On the other hand, root replacement, either using a valve-graft conduit (CRR) or valve-sparing (VSRR), aims to permanently treat aortic pathology, but at the expense of a higher surgical complexity and a prolonged operative time [2]. This study sought to compare short-term and mid-term hemostatic properties following root preservation with root replacement aortic repair in the context of treating ATAAD, thus assessing the hypothesis of non-inferiority of root preservation compared to root replacement.

## 2. Materials and Methods

### 2.1. Search Strategy and Article Selection

We conducted the present meta-analysis in accordance with a protocol agreed upon by all authors and following the Preferred Reporting Items for Systematic Reviews and Meta-Analyses (PRISMA) [3,4]. The PRISMA Checklist 2020 is demonstrated in Table S1. A thorough literature search was performed in Pubmed (Medline), Scopus (ELSEVIER), and Cochrane Central Register of Controlled Studies (CENTRAL) (last search: 18 September 2024). The following terms were employed in every possible combination: “root-sparing”, “root-preserving”, “root replacement”, “valve-sparing”, “bentall”, “david”, “yacoub”, “acute type a aortic dissection”, “ATAAD”, “aortic repair”, “hemostasis”, “bleeding”. We employed the following inclusion criteria: original reports with ≥ 50 patients, published from 2000 to 2024, written in English, conducted on human subjects, and reporting comparative outcomes of patients undergoing aortic repair for ATAAD with either the root-preserving or root replacement surgical technique. Duplicate articles were excluded. The reference lists of all included articles were also reviewed for additional studies. Two reviewers (DEM, AAR) extracted data from the included studies, working independently. Any discrepancies between the investigators were discussed with the senior author (TA) to include articles that best matched the criteria until consensus was reached.

### 2.2. Data Extraction and Endpoints

Regarding each eligible study, we extracted data on patient baseline characteristics, operative details, and perioperative outcomes. Reoperation for bleeding, the intraoperative transfusion of red blood cells (RBCs), the strategy of hemostatic sealing of the anastomosis in root repair following the reapproximation of the dissected layers of the aortic wall (with/without biological glue), and operational mortality were the primary endpoints. Operative mortality was defined as the 30-day or in-hospital mortality following surgery. Our original aim was also to assess the hemostasis time defined by the difference of total operation time minus cardiopulmonary bypass (CPB) time. Nonetheless, there was not enough available data for this certain evaluation. The incidence of postoperative acute kidney injury (AKI), prolonged ventilation, a cerebrovascular accident (CVA), the length of the hospital stay (LOS), and the overall survival (OS) and reintervention-free OS at 1 and 5 years postoperatively were the secondary endpoints. Studies with missing data were excluded in the related analyses and no imputation methods were used.

### 2.3. Subgroup and Sensitivity Analyses on Primary Endpoints

To validate our outcomes and to assess concerns regarding the consistency of outcome reporting across different studies and institutions, we performed an additional sensitivity analysis using the leave-one-out method. The leave-one-out method involves performing a meta-analysis on each subset of the studies obtained by leaving out exactly one study.

### 2.4. Quality and Publication Bias Assessment

The Newcastle–Ottawa Quality Assessment Scale (NOS) [5] was used as an assessment tool to evaluate the quality of the included non-Randomized Controlled Trials (RCTs). The scale’s range varies from zero to nine stars, and studies with a score equal to or higher than five were considered to have adequate methodological quality. The Risk of Bias in Non-Randomized Studies of Interventions tool (ROBINS-I) was also systematically used to assess the included studies for risk of bias [6]. No RCTs were identified in the literature to be included in the current meta-analysis. Two reviewers (DEM, AAR) rated the studies independently and a final decision was reached by consensus. Funnel plots were also visually inspected for the assessment of potential publication bias.

## 3. Results

### 3.1. Search Strategy and Patient Demographics

**Figure 1:**
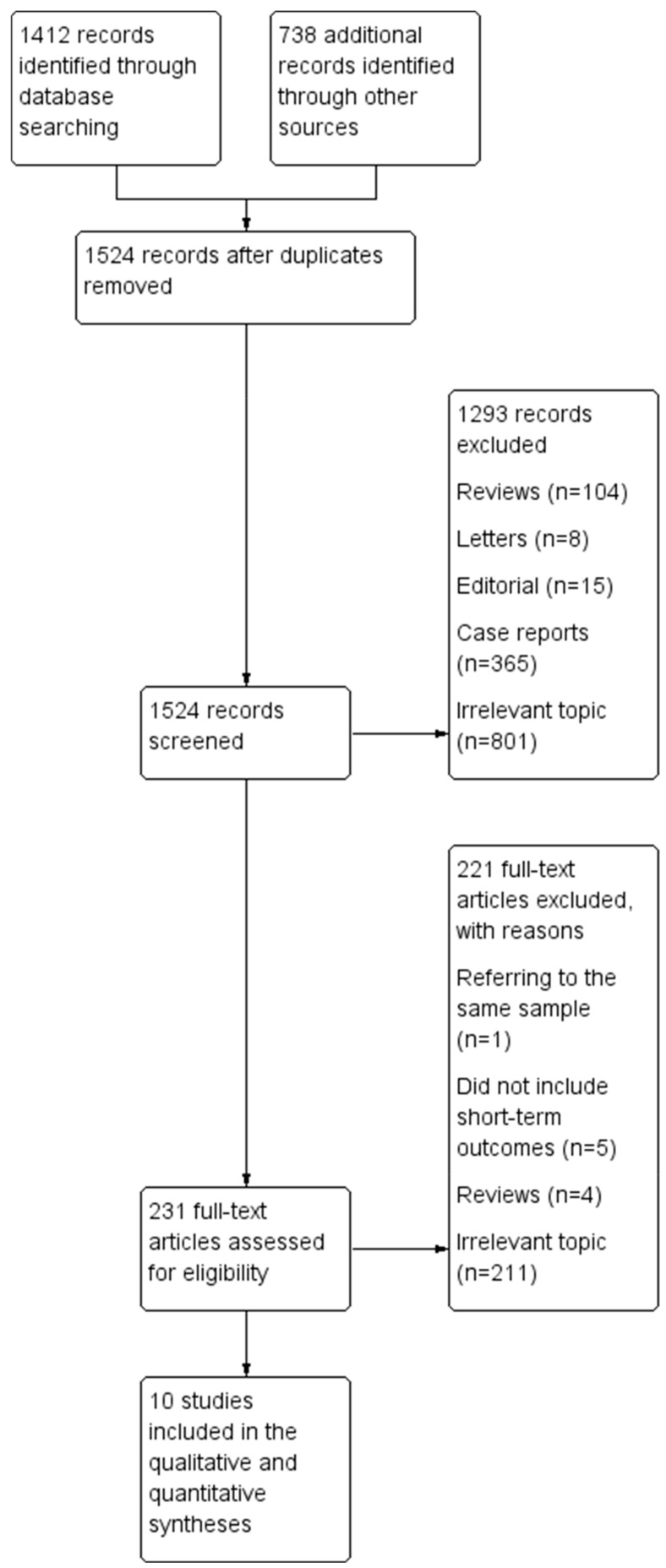
Literature search trial flow.

The flow diagram regarding the search strategy is shown in Figure 1 and the Prisma Checklist 2020 (Supplementary Materials). The main reason of article exclusion during the full text assessment was the irrelevance of their topic to our study. The characteristics of the included studies are summarized in Table 1. Among the 2150 articles in Pubmed, CENTRAL, and Scopus that were originally retrieved, 10 studies [7,8,9,10,11,12,13,14,15,16] were included in the qualitative and the quantitative synthesis. The level of agreement between the two reviewers was “substantial” (kappa = 0.80; 95% CI: 0.63, 0.97). The study design was retrospective in all studies. Risk adjustment was performed in three studies [7,9,13]. No RCTs were included in the current meta-analysis. The included studies were conducted in the USA [7,8,11,15,16], Italy [9], France [10], and China [13], and two were multinational [12,14]. The studies were published between 2014 and 2024. The total sample size was 6850 patients (RP: 4389; RR: 2461). Baseline characteristics, clinical presentation, and intraoperative parameters are shown in Table 1, Table 2, Table 3 and Table 4. Patients in the RR group had a higher rate of females, Marfan syndrome, aortic valve insufficiency, bicuspid aortic valve, previous cardiac surgery, and concomitant coronary artery bypass grafting, while patients in the RP group were older. The consistency of the definition of the primary endpoints (“operative mortality” and “reoperation for bleeding”) was high among the included studies and no difference was reported by the sensitivity analysis. All comparative outcomes are presented in Table 5.

### 3.2. Primary Endpoints

**Figure 2:**
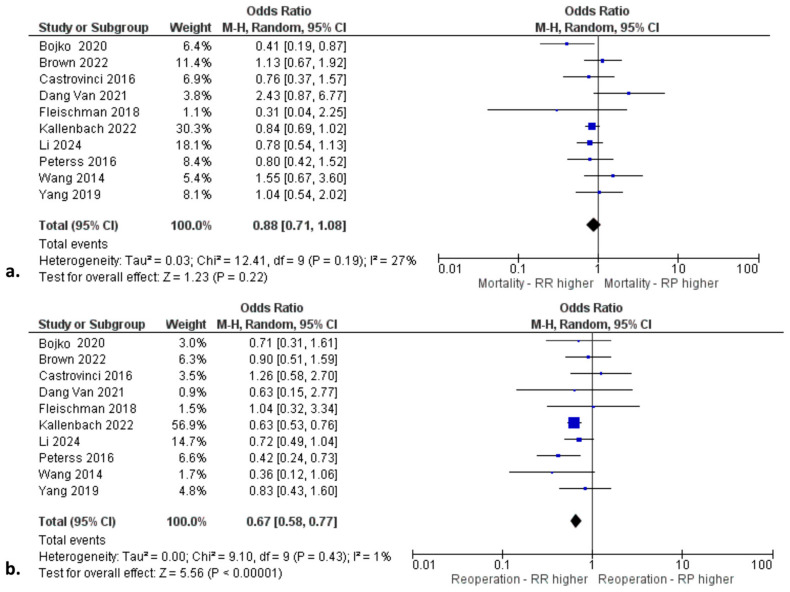
Forest plots for (**a**) operative mortality and (**b**) reoperation for bleeding [7,8,9,10,11,12,13,14,15,16].

The transfusion of RBCs (WMD: 1.00; 95% CI: −1.41, −0.59; *p* < 0.01) and the incidence of reoperation for excessive bleeding (OR: 0.67; 95% CI: 0.58, 0.77; *p* < 0.01) were significantly higher in the RR group. Most of the included studies did not use biological glue in order to limit the risk of anastomotic pseudoaneurysm formation. Instead, they used either the placement of polyester felt between the dissected layers as neomedia (“felt sandwich”), or the inversion of the adventitia, or a running circumferential suture with no use of surgical technical adjuncts. In fact, in one study [16], the use of biological glue was abandoned in all cases after 2005, based on the reported high incidence of anastomotic pseudoaneurysms in their earlier cases. In only one study [10], authors systematically implemented the use of biological glue. In that study [10], two patients (2.2%) underwent reoperation for an anastomotic pseudoaneurysm and both died following reoperation due to multiorgan failure. In addition, no significant difference was reported between the two groups regarding operative mortality (OR: 0.88; 95% CI: 0.71, 1.08; *p* = 0.22). Figure 2a shows the forest plot regarding operative mortality and Figure 2b shows the forest plot regarding the incidence of reoperation for excessive bleeding. The primary endpoints had low heterogeneity, thus limiting the potential publication bias (Table 5).

### 3.3. Secondary Endpoints

The forest plots for the secondary endpoints are presented in Figure S1. Both surgical approaches were associated with similar outcomes in terms of incidence of postoperative AKI, prolonged ventilation, CVA, and LOS. In addition, no difference was found between RP and RR regarding OS and reoperation-free survival at one and five years postoperatively. The main indications for long-term reoperation were endocarditis, a root abscess, severe aortic insufficiency, and an anastomotic pseudoaneurysm.

### 3.4. Sensitivity Analyses

No difference was found when we performed the leave-one-out sensitivity analysis regarding the primary and secondary outcomes.

### 3.5. Quality and Publication Bias Assessment

**Figure 3:**
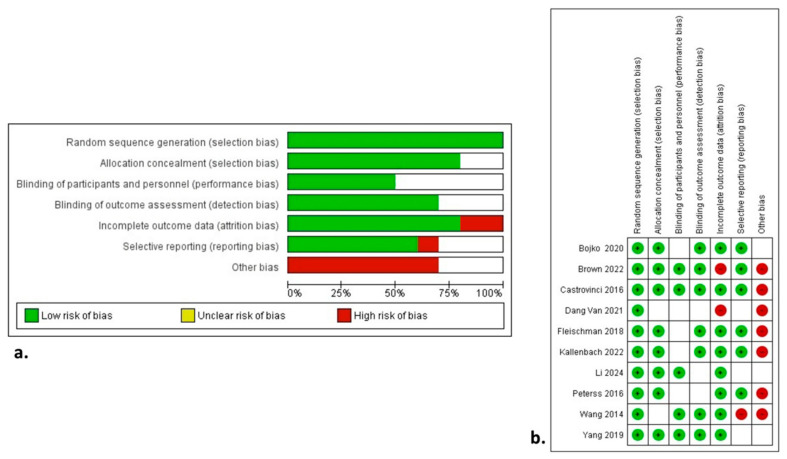
Risk of Bias in Non-Randomized Studies of Interventions with (**a**) summary plot and (**b**) traffic lights [7,8,9,10,11,12,13,14,15,16].

The NOS assessment of quality for all studies is shown in Table 1. Figure 3 demonstrates the qualitative assessment of the studies according to the ROBINS-I tool. The authors’ main concerns were mainly related to biases associated with the outcome data and selective reporting. The primary endpoints were associated with low heterogeneity. Most of the secondary endpoints were related to low heterogeneity. In contrast, the incidence of postoperative AKI, LOS, and reoperation-free OS were associated with high heterogeneity. The main factors affecting and increasing heterogeneity in these variables are the level of expertise, the volume of cases, and the differences in operation setting, along with the differences in the perioperative pathway protocols among different institutions. Funnel plots (Figure S2) seemed asymmetrical, with studies being absent from either the top or bottom of the graph, thus suggesting certain publication bias. For instance, there is the possibility of smaller studies with non-significant findings being less likely to be published. Nonetheless, we believe that the relatively small number of the included studies was the main reason for the reported asymmetry, and the validity of our outcomes was supported by the implementation of the Random-Effects model, the generally low heterogeneity, and the sensitivity analysis.

## 4. Discussion

The current study identified ten articles comparing the short-term and mid-term outcomes of aortic root preservation versus root replacement for patients with ATAAD as two alternative treatment strategies. The present study included a total of 6850 patients with a focus on the hemostatic properties of the two approaches. Given the lack of a randomized trial, the findings derived by the present pooled analysis provide the best currently available level of evidence on this topic. According to our total cohort analysis, RP was associated with fewer units of transfused RBCs and a lower incidence of reoperation for excessive bleeding. In addition, most studies did not use biological glue in root repair to avoid a anastomotic pseudoaneurysm. RP and RR were associated with similar operative mortality, overall survival, and reoperation-free survival at one and five years postoperatively. These findings were further validated by the sensitivity analysis using the leave-one-out method.

ATAAD represents a serious cardiac surgical emergency associated with high morbidity and mortality. The challenging surgical treatment is more complex in the presence of a concomitant existence of aortic root pathology. In this context, there are two main alternative surgical strategies for aortic repair, the more conservative root-preserving and the more aggressive root replacement aortic repair. In theory, both strategies have certain advantages. The root preservation avoids the manipulation of coronary arteries, thus potentially reducing cardiopulmonary and ischemic times [2]. In fact, according to our outcomes, RP demonstrated significantly lower CPB and cross-clamp times. On the other hand, root replacement might provide a definitive stabilization of the root, thus reducing the need for late reinterventions, thereby improving overall survival. Nonetheless, according to our findings, there was no significant difference between the two approaches in terms of overall and reoperation-free survival. Given concerns about long-term durability of the aortic root after root preservation and the lack of an RCT addressing this issue, it was critical to pool the available data to allow a direct comparison of overall survival and the need for early and mid-term reoperation. Our results support the non-inferiority of RP compared to RR in terms of operative mortality, as well as overall and reoperation-free survival.

A major concern regarding the RP strategy is its durability over time in terms of reoperation incidence. According to our analysis, the RP strategy is superior to RR regarding the incidence of reoperation for excessive bleeding. This finding is significant because previous studies have shown conflicting results. In fact, some studies suggested that RR is associated with a higher incidence of reoperation compared to RP [15], while other studies demonstrated similar outcomes [16]. The validity of our outcomes was also confirmed by the sensitivity analyses. These outcomes suggest the superior short-term hemostatic traits of the root-preserving approach. Certain previous imaging studies have demonstrated a higher risk of aortic disease progression in patients treated with a root-preserving approach [17,18]. According to our findings, the reoperation-free survival was similar during a 5-year follow-up period. The main indications for late reoperation were endocarditis, a root abscess, severe aortic insufficiency, and a root pseudoaneurysm. In the same context, some important predictors of aortic disease progression following RP aortic repair and reoperation have been suggested, such as the existence of connective tissue diseases, the aortic root diameter, the involvement of three sinuses, and the degree of aortic insufficiency [17,18].

Another important point we would like to further stress is related to blood transfusion as a metric of hemostatic traits for each approach. According to our findings, the number of transfused units of RBCs was significantly higher in the root replacement group. This metric is of great importance because, in ATAAD cases where bleeding occurs, a large number of blood products are inevitably transfused, thus triggering an avalanche of adverse events, such as impaired coagulation, along with electrolyte and acid–base impairments, which affect the patient’s prognosis [19]. In fact, ATAAD surgery is characterized by some distinct attributes mainly related to intraoperative hypothermia and total circulatory arrest, the coagulopathic state of these patients, along with the potential preoperative load with dual antiplatelet agents due to a misdiagnosis. Perhaps, the surgical technique plays a critical role in achieving adequate hemostasis through a proper false lumen obliteration and the construction of a resistant anastomosis. In this context, certain mechanistic characteristics of root replacement might contribute to the lower transfusions and reoperations for bleeding. Such characteristics are (1) the higher complexity of surgery, (2) increased dissection that includes the root and often reimplantation of coronary arteries, and (3) longer CPB and cross-clamp time, which contribute to (4) coagulopathy.

Certain hemostatic protocols have been proposed for the prevention and management of perioperative bleeding [19]. Different techniques have been proposed for this purpose, such as the reapproximation and reinforcement of the dissected aortic wall layers using Teflon felts (sandwich technique), the use of BioGlue for false lumen obliteration, or the use of the adventitia inversion, and neomedia formation technique [19]. Most of the included studies employed the Teflon sandwich technique and did not use biological glue to avoid the risk of an anastomotic pseudoaneurysm. Another surgical approach implements the local compressive maneuver [19]. This technique includes (1) the creation of a closed and pressurized space around the graft without compressing the heart, (2) meticulous hemostasis of the sternum and mediastinum, (3) transfer to the intensive care unit for hemodynamic and coagulative stabilization, and (4) closure in a second-look operation. Other hemostatic approaches suggest the implementation of alternatives to transfusion (e.g., fibrin, tranexamic acid, epsilon aminocaproic acid, etc.), and topical hemostatic agents [19]. Unfortunately, the included studies did not provide enough data to perform additional subgroup analyses comparing different hemostatic approaches. This might be an interesting topic of future studies.

There are certain limitations in the current study, mainly attributed to the inherent limitations of the included studies. First of all, no RCTs were included, thus posing a significant limitation. In fact, it is difficult to perform an RCT on this topic mainly due to ethical restrictions. Nonetheless, the lack of RCTs raises the value of the present meta-analysis since it provides the best available level of evidence and more solid outcomes compared to the individual studies. Although the majority of the studies were retrospective in nature, three of them provided risk-adjusted analyses. Furthermore, the incorporated studies are related to potential biases regarding differences in baseline characteristics between the two groups, the outcome data, and selective reporting. The severity of pathology of ATAAD might pose another important risk of bias. In fact, patients in the RP group may have had less severe pathology of ATAAD compared to the RR group, such as less extension of the dissection into the root, thus posing a certain risk of bias. Perhaps, a future well-designed RCT is necessary to minimize this risk and validate our outcomes. Moreover, the differences among institutions regarding the selection criteria, the surgeons’ expertise, and the perioperative management pose additional limitations. Cardiopulmonary bypass and cross-clamp time were associated with high heterogeneity, thus suggesting differences regarding intraoperative techniques among different centers. For this reason, we employed a Random-Effects model to perform the analysis of the endpoints and we also conducted a sensitivity analysis using the leave-one-out method. The low heterogeneity of the primary outcomes and most of the secondary endpoints demonstrates the validity of our findings, which was further confirmed by the sensitivity analysis.

On the other hand, the strengths of this study include the well-defined data-extraction protocol, the clear inclusion–exclusion criteria, the literature search in three distinct databases, the quality assessment of the included studies, and the detailed presentation of the results of data extraction and analyses, along with the sensitivity analysis that was performed.

## 5. Conclusions

In the context of patients undergoing aortic repair for ATAAD, root preservation without the use of biological glue during root repair is associated with enhanced hemostatic traits compared to the root replacement approach, a finding that should be further validated by a future RCT. In fact, the number of transfused units of RBCs and the incidence of reoperations for bleeding were higher in the RR group. Nonetheless, both techniques are similarly safe in terms of operative mortality and mid-term overall and reoperation-free survival. A future well-designed RCT should further validate our outcomes.

## Figures and Tables

**Table 1:** Baseline characteristics of the studies finally included in the meta-analysis.

Study ID, Year	Study Design	Patients, n RP/RR	Age, Years RP/RR	Female Sex, % RP/RR	Kidney Disease, % RP/RR	Previous CVA, % RP/RR	Marfan, % RP/RR	Previous CS, % RP/RR	Bicuspid AV, % RP/RR	AV Insufficiency, % RP/RR	NOS
Bojko 2020 [7]	R-RA	131/131	56 (48–66)/54 (44–66)	21/22	N/A	7/7	0/0	11/12	8/21	65/61	8
Brown 2022 [8]	R	370/231	63 ± 12/59 ± 15	42/37	N/A	N/A	N/A	10/16	2/8	34/54	6
Castrovinci 2016 [9]	R-RA	82/82	62 ± 12/63 ± 10	22/29	4/5	2/5	0/0	3/2	1/2	N/A	7
Dang Van 2021 [10]	R	118/46	65 ± 11/52 ± 16	40/13	2/7	N/A	2/9	4/4	3/11	44/66	6
Fleischman 2018 [11]	R	148/47	62 ± 13/57 ± 16	31/21	9/6	7/6	1/6	11/26	N/A	14/51	7
Kallenbach 2022 [12]	R	2425/957	63 ± 13/CR: 58 ± 13; VS: 54 ± 14	39/CR: 32; VS: 26	Ν/A	N/A	2/CR: 5; VS: 9	N/A	N/A	26/CR: 57; VS: 11	7
Li 2024 [13]	R-RA	447/628	57 (52–64)/57 (51–64)	35/31	N/A	N/A	N/A	N/A	N/A	16/18	7
Peterss 2016 [14]	R	249/89	62 ± 13/56 ± 13	34/24	N/A	6/7	N/A	6/7	1/16	35/72	6
Wang 2014 [15]	R	112/66	65 ± 14/53 ± 17	17/10	N/A	5/11	1/8	5/11	6/24	30/65	6
Yang 2019 [16]	R	307/184	61 (52–59)/56 (44–67)	36/21	6/10	2/3	0/7	7/11	3/19	27/65	7

The Newcastle–Ottawa Scale (NOS) for assessing the quality of non-randomized studies. The highest-quality studies are awarded up to 9 stars. Abbreviations: RP = Root Preservation; RR = Root Repair; R = Retrospective; P = Prospective; RA = Risk Adjusted; N/A = Not Available; DM = Diabetes Mellitus; CVA = Cerebrovascular Accident; CS = Cardiac Surgery; AV = Aortic Valve; CR = Conduit Replacement; VS = Valve-sparing.

**Table 2:** Patients’ clinical presentation at admission.

Study ID, Year	Type of Malperfusion, % RP/RR					Tamponade, Rupture, or Shock, % RP/RR
	Cerebral	Coronary	Visceral	Renal	Iliofemoral	
Bojko [7]	26/14	6/40	23/11	3/0	34/29	24/24
Brown [8]	12/13	5/10	6/5	5/7	14/14	34/27
Castrovinci [9]	2/5	N/A	1/1	4/5	N/A	8/11
Dang Van [10]	N/A	N/A	5/0	N/A	13/9	25/22
Fleischman [11]	7/6	N/A	7/9	N/A	9/11	26/25
Kallenbach [12]	13/9	8/18	7/6	9/10	7/7	21/21
Li [13]	27/23					N/A
Peterss [14]	16/13	18/21				20/20
Wang [15]	N/A	N/A	N/A	N/A	N/A	N/A
Yang [16]	4/5	2/5	10/10	7/8	10/7	9/12

Abbreviations: RP = Root preservation; RR = Root repair; N/A = Not available.

**Table 3:** Intraoperative parameters.

Study ID, Year	Arch Replacement, % RP/RR		Type of Arterial Cannulation, % RP/RR				Cerebral Perfusion, % RP/RR		Concomitant CABG, % RP/RR
	Hemi-Arch	Total Arch	Axillary	Femoral	Axillary and Femoral	Aortic Arch	ACP	RCP	
Bojko [7]	91/92	9/8	N/A	N/A	N/A	N/A	N/A	N/A	4/21
Brown [8]	63/65	37/35	10/8	5/6	N/A	85/81	N/A	N/A	13/17
Castrovinci [9]	71/71	11/11	N/A	N/A	N/A	N/A	N/A	N/A	27/95
Dang Van [10]	45/35	4/7	N/A	N/A	N/A	N/A	25/42	56/33	4/2
Fleischman [11]	N/A	N/A	64/55	9/9	24/30	N/A	80/75	15/25	7/30
Kallenbach [12]	46/49	14/16	42/45	18/6	N/A	5/2	70/53	2/1	N/A
Li [13]	12/11	79/82	N/A	N/A	N/A	N/A	88/90	12/10	8/7
Peterss [14]	81/84	16/12	36/34	57/63		6/3	39/53	16/15	10/15
Wang [15]	70/95	30/5	N/A	N/A	N/A	N/A	N/A	N/A	N/A
Yang [16]	58/64	8/4	N/A	N/A	N/A	N/A	32/27	36/41	4/9

Abbreviations: RP = Root Preservation; RR = Root Repair; N/A = Not Available; ACP = Antegrade Cerebral Perfusion; RCP = Retrograde Cerebral Perfusion; CABG = Coronary Artery Bypass Grafting.

**Table 4:** Summary of baseline characteristics.

Baseline Characteristics	Arms	WMD/OR *	95% CI	*p*-Value	Heterogeneity	
					I2	*p*-Value
Age	11	5.05	2.93, 7.916	<0.01	96%	<0.01
Female Ratio	11	0.62	0.42, 0.93	0.02	79%	<0.01
DM	6	1.14	0.84, 1.57	0.40	0%	0.93
Kidney Disease	4	0.49	0.20, 1.18	0.11	48%	0.12
Previous CVA	6	0.73	0.46, 1.16	0.18	0%	0.54
Marfan	8	0.23	0.14, 0.39	<0.01	28%	0.23
Previous CS	7	0.64	0.48, 0.86	<0.01	0%	0.65
Bicuspid AV	6	0.20	0.12, 0.32	<0.01	29%	0.22
AV Insufficiency	9	0.36	0.25, 0.54	<0.01	88%	<0.01
Tamponade/Rupture	9	1.04	0.90, 1.19	0.60	0%	0.75
Concomitant CABG	9	0.33	0.15, 0.73	<0.01	89%	<0.01

Abbreviations: CPB = Cardiopulmonary Bypass; AKI = Acute Kidney Disease; CVA = Cerebrovascular Accident; LOS = Length Of Stay; OS = Overall Survival; WMD = Weighted Mean Difference; OR = Odds Ratio; CI = Confidence Interval. * Mantel–Haenszel (M-H) method was employed for categorical variables and Inverse Variance (IV) for continuous variables.

**Table 5:** Summary of primary and secondary endpoints.

Endpoints	Arms	WMD/OR *	95% CI	*p*-Value	Heterogeneity	
					I2	*p*-Value
Operative mortality	10	0.88	0.71, 1.08	0.22	27%	0.19
Transfusion of RBCs	2	−1.00	−1.41, −0.59	<0.01	0%	1.00
Reoperations for bleeding	10	0.67	0.58, 0.77	<0.01	1%	0.43
Aortic cross-clamp time	11	−54.18	−66.24, −42.12	<0.01	98%	<0.01
CPB time	11	−48.93	−55.88, −41.98	<0.01	86%	<0.01
Postoperative AKI	10	1.24	0.92, 1.69	0.16	69%	<0.01
Prolonged ventilation	7	1.02	0.78, 1.34	0.89	33%	0.17
CVA	10	0.94	0.81, 1.10	0.47	0%	0.55
LOS	7	−0.13	−1.80, 1.54	0.88	95%	<0.01
1-year OS	6	1.01	0.76, 1.33	0.96	52%	0.06
5-year OS	8	0.87	0.72, 1.05	0.15	25%	0.23
1-year reoperation-free OS	3	0.82	0.59, 1.13	0.22	31%	0.23
5-year reoperation-free OS	7	0.86	0.66, 1.13	0.27	61%	0.02

Abbreviations: CPB = Cardiopulmonary Bypass; AKI = Acute Kidney Disease; CVA = Cerebrovascular Accident; LOS = Length Of Stay; OS = Overall Survival; RBCs = Red Blood Cells; OR = Odds Ratio; WMD = Weighted Mean Difference; CI = Confidence Interval. * WMD was calculated for continuous outcomes and OR for categorical outcomes.

## Data Availability

Data are available from the authors upon reasonable request.

## Supplementary Materials

The following supporting information can be downloaded at: https://www.mdpi.com/article/10.3390/life14101255/s1, Figure S1: Forest plots for secondary outcomes; Figure S2: Funnel plots for primary and secondary outcomes. Table S1: PRISMA Checklist.
