# Supplementary material for: Hemostatic Properties of Aortic Root Preservation versus Root Replacement for Acute Type A Aortic Dissection: A Pooled Analysis
Source: Life (Basel). 2024 Oct 1;14(10):1255. doi: 10.3390/life14101255 (PMC11508654; doi:10.3390/life14101255)
Supplement: Supplementary file 1 [file life-14-01255-s001.zip › Figure S1.pdf]

Study or Subgroup	Weight	Odds Ratio M-H, Random, 95% CI
Bojiko 2020	9.1%	0.57 [0.30, 1.11]
Brown 2022	10.6%	1.57 [0.91, 2.73]
Castrovinci 2016	9.5%	1.05 [0.56, 1.98]
Dang Van 2021	8.0%	3.29 [1.53, 7.09]
Fleischman 2018	5.9%	2.70 [0.99, 7.34]
Kallenbach 2022	14.0%	1.12 [0.84, 1.49]
Li 2024	14.1%	0.83 [0.62, 1.10]
Peterss 2016	10.2%	0.63 [0.36, 1.12]
Wang 2014	9.6%	1.88 [1.01, 3.51]
Yang 2019	9.0%	1.96 [1.00, 3.87]
**Total (95% CI)**	**100.0%**	**1.24 [0.92, 1.69]**
Total events		

Heterogeneity: Tau² = 0.15; Chi² = 29.45, df = 9 (P = 0.0005); I² = 69%
Test for overall effect: Z = 1.40 (P = 0.16)

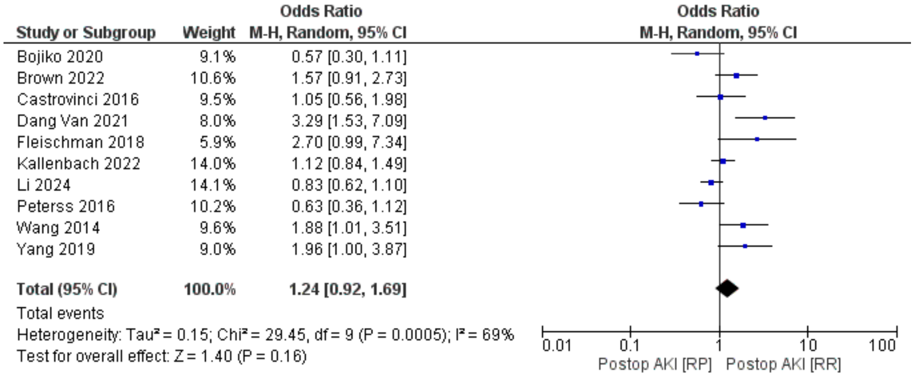

a.

Study or Subgroup	Weight	Odds Ratio M-H, Random, 95% CI
Bojiko 2020	2.3%	1.15 [0.41, 3.27]
Brown 2022	2.9%	2.14 [0.85, 5.42]
Castrovinci 2016	5.2%	1.36 [0.68, 2.71]
Dang Van 2021	1.0%	2.04 [0.43, 9.68]
Fleischman 2018	2.1%	0.88 [0.30, 2.58]
Kallenbach 2022	61.4%	0.87 [0.71, 1.06]
Li 2024	9.8%	1.01 [0.61, 1.67]
Peterss 2016	5.4%	0.85 [0.43, 1.66]
Wang 2014	1.8%	1.86 [0.57, 6.02]
Yang 2019	8.0%	0.77 [0.44, 1.33]
**Total (95% CI)**	**100.0%**	**0.94 [0.81, 1.10]**
Total events		

Heterogeneity: Tau² = 0.00; Chi² = 7.81, df = 9 (P = 0.55); I² = 0%
Test for overall effect: Z = 0.73 (P = 0.47)

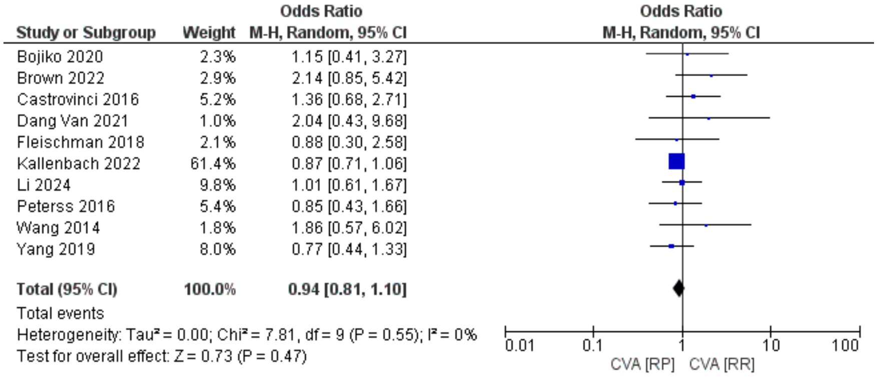

b.

Study or Subgroup	Weight	Mean Difference IV, Random, 95% CI
Dang Van 2021	16.7%	-1.00 [-1.63, -0.37]
Fleischman 2018	16.0%	-3.00 [-4.08, -1.92]
Kallenbach 2022	12.4%	-0.40 [-2.88, 2.08]
Kallenbach 2022	14.0%	2.10 [0.20, 4.00]
Li 2024	16.5%	3.00 [2.20, 3.80]
Peterss 2016	7.7%	-5.10 [-9.59, -0.61]
Yang 2019	16.8%	1.00 [0.46, 1.54]
**Total (95% CI)**	**100.0%**	**-0.13 [-1.80, 1.54]**

Heterogeneity: Tau² = 4.26; Chi² = 111.85, df = 6 (P < 0.00001); I² = 95%
Test for overall effect: Z = 0.15 (P = 0.88)

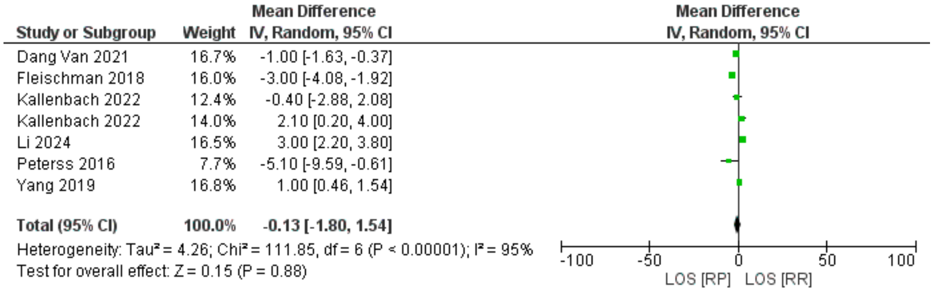

c.

Study or Subgroup	Weight	Odds Ratio M-H, Random, 95% CI
Bojiko 2020	17.8%	0.82 [0.50, 1.36]
Castrovinci 2016	13.2%	0.66 [0.35, 1.25]
Dang Van 2021	8.6%	1.53 [0.66, 3.52]
Fleischman 2018	3.7%	0.95 [0.25, 3.66]
Peterss 2016	18.3%	0.73 [0.45, 1.19]
Wang 2014	13.6%	1.50 [0.81, 2.77]
Yang 2019	24.8%	1.35 [0.94, 1.96]
**Total (95% CI)**	**100.0%**	**1.02 [0.78, 1.34]**
Total events		

Heterogeneity: Tau² = 0.04; Chi² = 8.99, df = 6 (P = 0.17); I² = 33%
Test for overall effect: Z = 0.13 (P = 0.89)

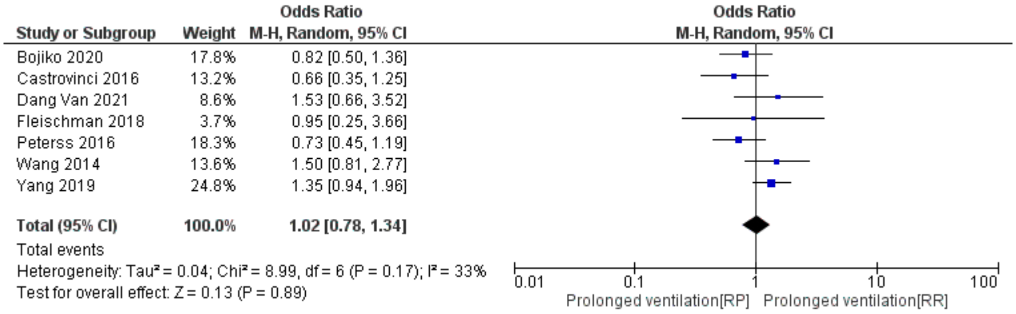

d.

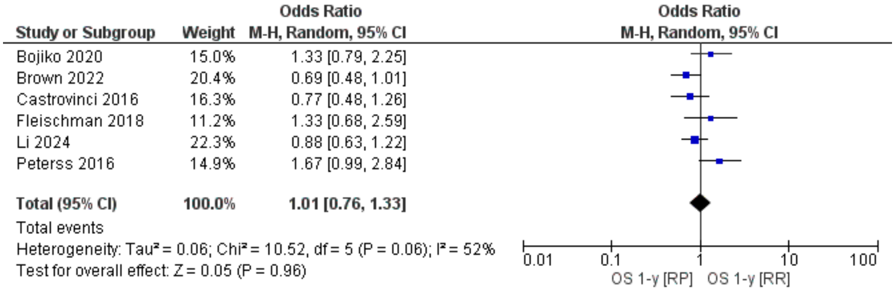

e.

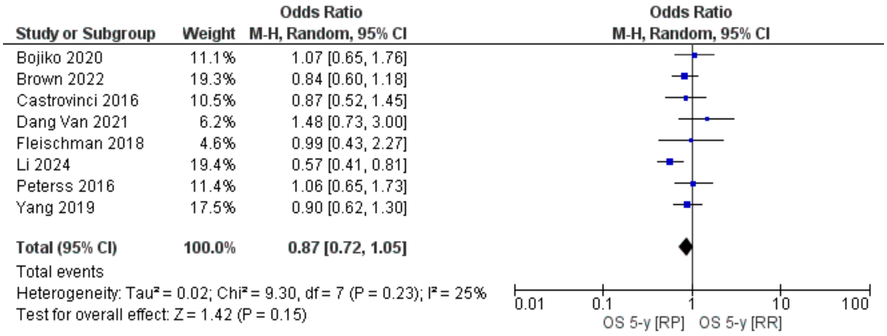

f.

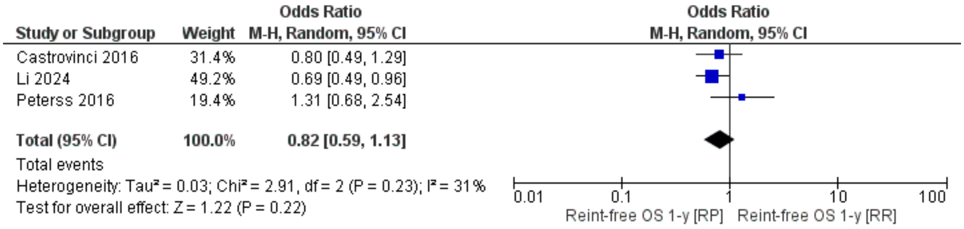

g.

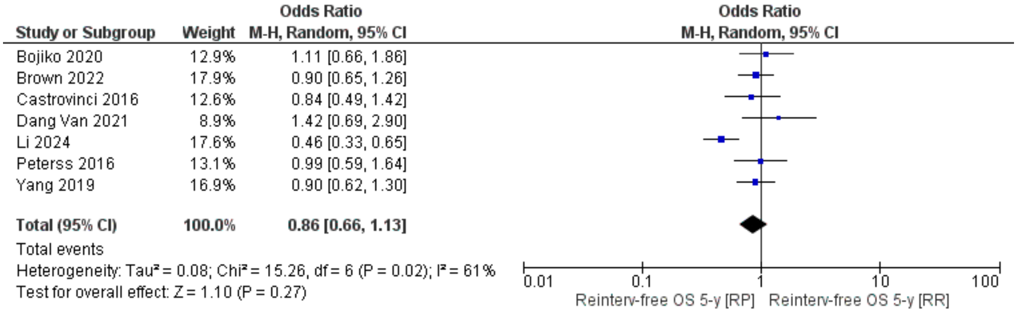

h.

Figure S1. Forest plots describing the differences in rates of (a) postoperative acute kidney disease, (b) cerebrovascular accident, (c) length of stay, (d) prolonged ventilation, (e) overall survival at 1-year postoperatively, (f) overall survival at 5-years postoperatively, (g) reintervention-free survival at 1-year postoperatively, (h) reintervention-free survival at 5-years postoperatively
