# Supplementary material for: Hemostatic Properties of Aortic Root Preservation versus Root Replacement for Acute Type A Aortic Dissection: A Pooled Analysis
Source: Life (Basel). 2024 Oct 1;14(10):1255. doi: 10.3390/life14101255 (PMC11508654; doi:10.3390/life14101255)
Supplement: Supplementary file 1 [file life-14-01255-s001.zip › Figure S2.pdf]

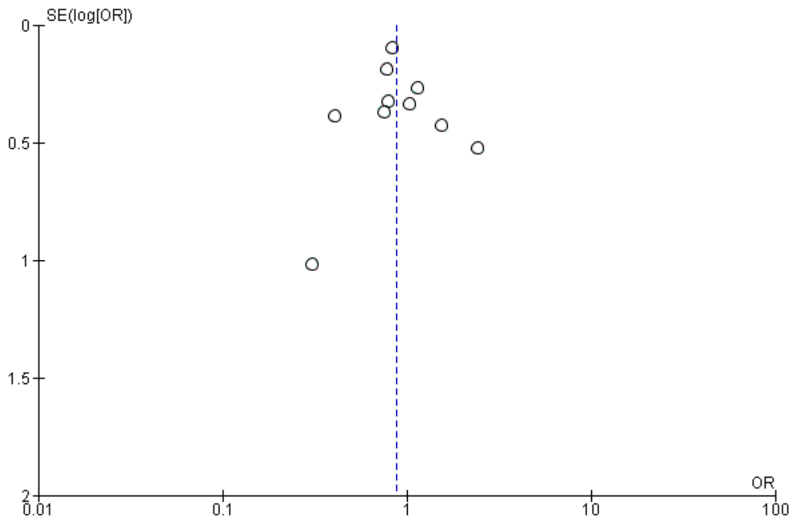
SE(log[OR])
0
0.5
1
1.5
2
OR
0.01
0.1
1
10
100

a.

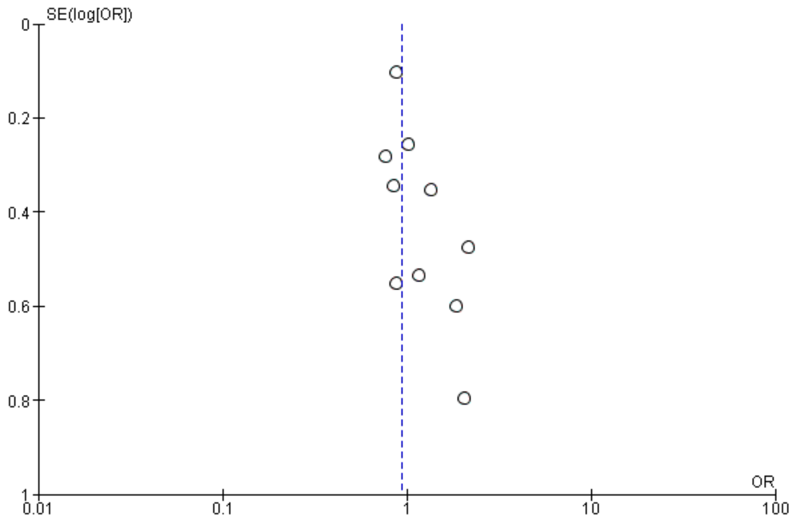
SE(log[OR])
0
0.2
0.4
0.6
0.8
1
OR
0.01
0.1
1
10
100

b.

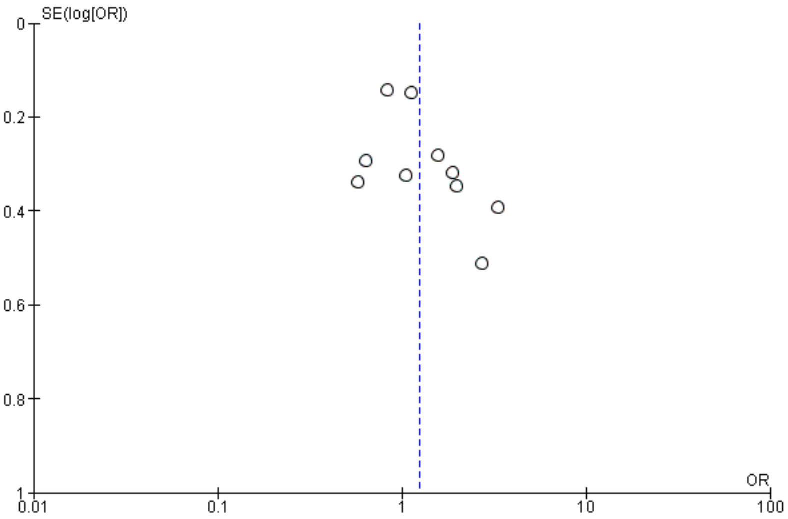

c.

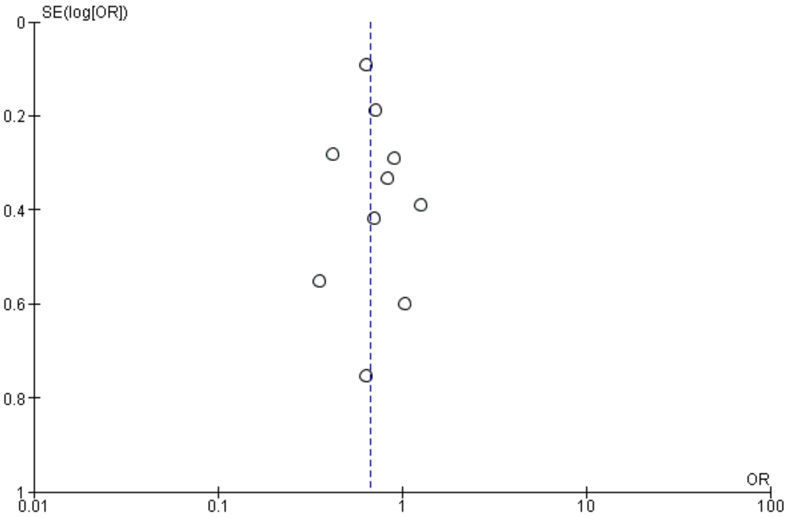

d.

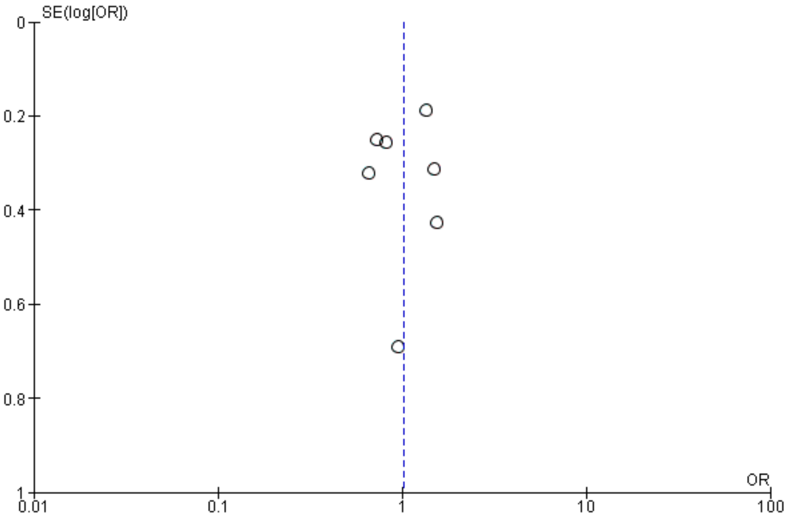

e.

Figure S2. Funnel plots regarding (a) operative mortality, (b) cerebrovascular accident, (c) postoperative acute kidney disease, (d) reoperation, (e) prolonged ventilation
